# MLDT: an open-source multilayer diffusion theory Python implementation

**DOI:** 10.1117/1.JBO.31.9.095006

**Published:** 2026-09-29

**Authors:** Thomas T. Livecchi, Mark C. Pierce, Steven L. Jacques

**Affiliations:** a Rutgers, The State University of New Jersey, Department of Biomedical Engineering, Piscataway, New Jersey, United States; b University of Washington, Department of Bioengineering, Seattle, Washington, United States

**Keywords:** radiative transport, diffusion theory, tissue reflectance, tissue modeling

## Abstract

**Significance:**

Diffuse reflectance spectroscopy and imaging are powerful noninvasive tools for quantifying tissue biomarkers such as hemoglobin, melanin, and water. A theoretical model of light propagation is required to interpret these reflectance measurements and translate them into quantitative biomarker concentrations. Although Monte Carlo (MC) simulations offer the gold-standard in accuracy, their high computational burden renders them impractical for real-time iterative inverse solving. Conversely, standard analytical approximations can be efficient but often oversimplify tissue as a semi-infinite homogeneous medium, leading to significant errors when analyzing layered structures.

**Aim:**

We aim to develop a generalized, computationally efficient forward model capable of rapidly predicting light transport in complex, multilayered tissue structures.

**Approach:**

We present a generalized derivation and open-source Python implementation of the P1 diffusion approximation for predicting diffuse reflectance from tissue with an arbitrary number of layers. This framework utilizes a vectorized banded matrix approach to solve the fluence rate equations efficiently. The model was validated against 400 randomized MC simulations encompassing architectures ranging from one to eight layers.

**Results:**

Diffuse reflectance spectra predicted by the generalized multilayer diffusion theory model show strong agreement with MC simulations in the diffusion regime ($\mu_{a}\ll \mu_{s}^{\prime }$), achieving a median spectral root mean square error of 0.0145 across the validation set. Computation times were over 5 orders of magnitude faster than GPU-accelerated MC, yielding a mean calculation time of 0.0087 s to generate a single predicted diffuse reflectance spectrum given a set of input optical properties and a speedup of ∼277,607×. Notably, validation results demonstrated that numerical error does not scale with structural complexity, indicating that multilayer architectures maintained high stability.

**Conclusions:**

We provide an accurate, computationally efficient, and generalized tool for modeling light transport in stratified media. By combining the ability of MC modeling to simulate complex tissue structures, with the speed of analytical diffusion methods, this tool facilitates real-time, depth-resolved quantitative tissue profiling. The full source code is made freely available to the community.

## 1 Introduction

Noninvasive optical diagnostics are increasingly utilized in clinical applications ranging from skin cancer detection to monitoring tissue hemodynamics. These techniques rely on solving the inverse problem of deducing physiological properties such as tissue oxygenation and melanin concentration from measured optical signals. To achieve this, a forward model is required that accurately relates a tissue’s optical properties (absorption coefficient $\mu_{a}$ and reduced scattering coefficient $\mu_{s}^{\prime }$) to its diffuse reflectance spectrum.

A fundamental challenge in tissue optics is accurately modeling the stratified architecture of biological tissues. Human skin, for example, is inherently multilayered, consisting of a thin, melanin-rich epidermis, a blood-rich dermis, and a subcutaneous fat layer.^1^^–^^3^ Functional near-infrared spectroscopy uses multilayer tissue models for scalp, skull, and brain tissue.^4^ Standard homogeneous models, which assume a semi-infinite uniform medium, are mathematically convenient but physically inadequate for such structures. They often fail to distinguish between surface absorption and deeper absorption, leading to cross-talk errors where changes in one layer fundamentally bias the recovered parameters of another.

In quantitative tissue optics, the relationship between light and matter is characterized by two distinct problems. The forward problem involves predicting the measurable reflectance spectrum based on a known set of underlying optical properties ($\mu_{a},\mu_{s}^{\prime }$). This requires a solution to the radiative transport equation (RTE) to simulate how light propagates through a specific geometry. Conversely, the inverse problem is the process of deducing those unknown physiological properties from the measured reflectance signals. Because the inverse problem is typically solved by iteratively adjusting parameters until the model’s output matches the measurement, the accuracy of the recovered properties is fundamentally dependent on the precision of the underlying forward model. These models range from fast analytical approximations to computationally intensive numerical simulations.

For applications where computational speed is paramount, the RTE can be simplified into an analytically solvable form. The most common approach is diffusion theory, which can be formally derived using the P1 approximation.^5^ The P1 approximation assumes that the radiance can be approximated by a linear combination of an isotropic component (the fluence rate) and a directional component (the flux). This simplification is valid in highly scattering media where the scattering coefficient is much larger than the absorption coefficient ($\mu_{s}^{\prime }\gg \mu_{a}$). Higher-order spherical harmonics approaches, such as the P3 approximation, have been developed for multilayer media to bridge the accuracy gap between the standard P1 approximation and Monte Carlo simulations.^6^

To address tissue heterogeneity, researchers have traditionally relied on stochastic Monte Carlo (MC) simulations and deterministic analytical approximations. Monte Carlo methods, particularly accelerated GPU-based implementations like MCX,^7^^–^^9^ are considered the gold standard for accuracy as they rigorously solve the RTE. However, despite hardware acceleration, MC remains computationally expensive for iterative inverse fitting routines that may require thousands of forward calls per measurement. Although look-up table approaches^10^^–^^12^ and machine learning^13^^–^^15^ surrogates have been employed to bypass this bottleneck, they often lack flexibility, requiring extensive retraining or regeneration when the model geometry changes.

Early work by Farrell et al.^2^ established the utility of the diffusion approximation for estimating the spatially resolved diffuse reflectance from semi-infinite media. This work utilized a dipole source model to simplify the relationship between optical properties and measured signals. Hielscher et al.^16^ and Kienle et al.^17^ proved that the partial-current boundary condition offers a much more accurate representation of photon migration than simpler models. This improvement is especially important for steady-state reflectance where the refractive index mismatch significantly impacts the light distribution near the surface. Although these advancements refined the accuracy of the diffusion approximation for uniform tissue volumes, they also introduced more complex math when internal boundaries are added.

To address the layered nature of biological tissue, researchers developed multilayer diffusion models to separate the epidermis and dermis. Early work by Keijzer et al.^18^ established that boundary conditions for surfaces and internal interfaces could be solved using numerical methods to account for refractive index mismatches. Later, Spott et al.^1^ further refined two-layer diffusion models using photon density waves. Although these models represented a major step forward, they often rely on solving transcendental equations or finite element meshes that can become unstable or computationally demanding as more layers are added. There is still a practical gap in the field because current analytical tools lack a unified and efficient way to scale from a few layers to many layers. Most existing software is either closed source or not optimized for the rapid iterative calls needed for inverse profiling. This work addresses that gap by providing a generalized matrix approach that remains stable and fast for any number of layers.

This work presents the derivation and validation of a generalized multilayer diffusion theory (MLDT) solver implemented in Python. By employing a vectorized matrix formalism, this solver extends the analytical diffusion solution to N layers with negligible computational overhead. The approach is validated across a wide range of optical properties and structural complexities against gold-standard Monte Carlo simulations to evaluate its forward predictive accuracy, numerical stability, and speed. The complete source code is provided as an open-source resource (https://github.com/ttlivecchi/mldt.git), enabling the biomedical optics community to easily integrate multilayer physics into inverse profiling workflows.

## 2 Methods

### 2.1 Physics

This derivation extends the two-layer diffusion model derived by Spott et al.^1^ to a generalized system of N stratified layers. By retaining the core boundary conditions used in the two-layer case, we formulate a recursive matrix approach that solves for the fluence rate across arbitrary geometries. For each layer i, the optical properties are the absorption coefficient ($\mu_{a,i}$) and the reduced scattering coefficient ($\mu_{s,i}^{\prime }$). We assume an incident light power $\Phi_{t}$ (which will factor out in reflectance calculations) and a uniform bulk refractive index $n_{r}$ for the tissue medium, assuming index-matched internal layers.

The first fundamental relationship of the diffusion approximation is the conservation of energy, which states that the rate of change of energy density within a volume is equal to the net flow of energy into or out of the volume plus any energy generated or absorbed within it. The second fundamental relationship of the diffusion approximation is Fick’s Law, which relates the net flow of energy (the flux) to the gradient of the energy density (the fluence rate).

Combining these two principles, the steady-state diffusion equation for the fluence rate ϕ(z), which describes the total radiant energy flowing across an infinitesimal sphere at position z, can be derived. For a homogeneous medium with a source q(z), the equation is

$$\nabla^{2}\phi -\frac{\phi }{\delta^{2}}=-\frac{q}{D}.$$ (1)

The key parameters of this equation are the transport coefficient $\mu_{\mathrm{tr}}$, the diffusion length D, and the penetration depth δ. The transport coefficient, $\mu_{\mathrm{tr}}$, is a combination of the absorption and reduced scattering coefficients, as shown in Eq. (2).

$$\mu_{\mathrm{tr}}=\mu_{a}+\mu_{s}^{\prime }.$$ (2)

The diffusion length, D, relates the flux to the fluence rate and is defined as

$$D=\frac{1}{3\mu_{\mathrm{tr}}}.$$ (3)

The penetration depth, δ, is the average distance a photon travels from the point of scattering to the point of absorption, and it is related to the transport and absorption coefficients by

$$\delta =\frac{1}{\sqrt{3\mu_{a}\mu_{\mathrm{tr}}}}.$$ (4)

For a collimated incident light beam, the source term q(z) is often approximated as light scattered out of the beam into diffuse light. Here, a distributed source term is implemented, which assumes an exponential decay in intensity with increasing distance from the surface.^1^^,^^3^^,^^5^ For layer i, this is

$$q_{i}(z)=\Phi_{t}\mu_{s,i}^{\prime }e^{-\mu_{\mathrm{tr},i}z^{\prime }}.$$ (5)

Here, $\Phi_{t}$ is the transmitted irradiance, representing the power per unit area of the incident beam that successfully enters the tissue after specular reflection. $z^{\prime }$ is the depth into the current layer. The total light field is decoupled into a collimated component and a diffuse component. The distributed source term $q_{i}(z)$ specifically represents the spatial rate at which the collimated incident beam undergoes its initial scattering events and transitions into diffuse, nearly isotropic light. Crucially, $\Phi_{t}$ represents the initial transmitted irradiance entering the surface of the first layer (z=0). As this collimated beam passes straight down through a multilayered medium, it undergoes exponential decay governed by the cumulative transport coefficients ($\mu_{\mathrm{tr}}$) and thicknesses of all overlying layers. Every coordinate along this decaying path acts as a local birth site for diffuse photons. Within any given layer, the linear superposition of these isotropic source elements yields a particular mathematical solution that shares the exponential decay profile of the driving collimated beam. When integrated with the boundary-driven diffuse waves that migrate back and forth due to mismatch reflections at internal and physical boundaries, this forms the complete framework of MLDT expanded in Sec. 2.2.

### 2.2 Generalized Solution for *N* Layers

Although two-layer models have been derived by Spott et al.^1^ and Keijzer et al.,^18^ biological tissues are often more complex. This section expands the diffusion approximation to an arbitrary number of layers, providing a more generalized solution based on multilayer diffusion theory (MLDT).

Consider a system with N layers. The first $N_{\mathrm{finite}}$ layers have finite thicknesses, and the last layer is semi-infinite. Thus, $N=N_{\mathrm{finite}}+1$. For each layer k (where k ranges from 0 to N−1), the thicknesses of the finite layers are $d_{k}$ for k from 0 to $N_{\mathrm{finite}}-1$. The absolute starting depth of layer k is $z_{k,\mathrm{start}}$.

The general solution for the fluence rate $\phi_{k}(z)$ in layer k (for 0≤k<N−1, the finite layers) is

$$\phi_{k}(z)=S_{0,k}e^{-\mu_{\mathrm{tr},k}(z-z_{k,\mathrm{start}})}+C_{k,\mathrm{decay}}e^{-(z-z_{k,\mathrm{start}})/\delta_{k}}+C_{k,\mathrm{grow}}e^{(z-z_{k,\mathrm{start}})/\delta_{.}}$$ (6)

The coefficients $C_{k,\mathrm{decay}}$ and $C_{k,\mathrm{grow}}$ represent the integration constants required to satisfy the boundary conditions at the top and bottom of each finite layer. In traditional semi-infinite models, only a single decaying exponential is used because the fluence must vanish at infinite depth. However, in a stratified system with N layers, light reflects and scatters at every internal interface. The $C_{k,\mathrm{decay}}$ term accounts for the portion of the diffuse intensity that decreases with depth, whereas the $C_{k,\mathrm{grow}}$ term accounts for the diffuse intensity that appears to increase as it reflects back from deeper boundaries toward the surface. By separating the solution into these two components for every finite layer, a matrix approach can account for the complex back and forth migration of photons between multiple interfaces. For the last layer (k=N−1, semi-infinite), the growing exponential term vanishes:

$$\phi_{N-1}(z)=S_{0,N-1}e^{-\mu_{\mathrm{tr},N-1}(z-z_{N-1,\mathrm{start}})}+C_{N-1,\mathrm{decay}}e^{-(z-z_{N-1,\mathrm{start}})/\delta_{N-1}}.$$ (7)

Here, $S_{0,k}$ is the coefficient for the particular solutions, accounting for attenuation of the incident beam as it reaches layer k:

$$S_{0,k}=\frac{\Phi_{t}\delta_{k}^{2}\mu_{s,k}^{\prime }}{D_{k}(1-\mu_{\mathrm{tr},k}^{2}\delta_{k}^{2})}\cdot (\overset{k-1}{\underset{i=0}{\prod }}e^{-\mu_{\mathrm{tr},i}d_{i}}),$$ (8)

where the product term is 1 for k=0 (the first layer). The term $e^{-\mu_{\mathrm{tr},i}d_{i}}$ accounts for the attenuation of the incident collimated beam as it passes through layer i.

The flux $j_{k}(z)$ in layer k is related to the fluence rate by

$$j_{k}(z)=-D_{k}\frac{d\phi_{k}}{dz}.$$ (9)

The derivatives of the fluence rates for finite layers are

$$\frac{d\phi_{k}}{dz}=-S_{0,k}\mu_{\mathrm{tr},k}e^{-\mu_{\mathrm{tr},k}(z-z_{k,\mathrm{start}})}-\frac{C_{k,\mathrm{decay}}}{\delta_{k}}e^{-(z-z_{k,\mathrm{start}})/\delta_{k}}+\frac{C_{k,\mathrm{grow}}}{\delta_{k}}e^{(z-z_{k,\mathrm{start}})/\delta_{k}}.$$ (10)

The derivative of the fluence rate for the last layer is

$$\frac{d\phi_{N-1}}{dz}=-S_{0,N-1}\mu_{\mathrm{tr},N-1}e^{-\mu_{\mathrm{tr},N-1}(z-z_{N-1,\mathrm{start}})}-\frac{C_{N-1,\mathrm{decay}}}{\delta_{N-1}}e^{-(z-z_{N-1,\mathrm{start}})/\delta_{N-1}}.$$ (11)

There are $2\times N_{\mathrm{finite}}+1$ unknown coefficients ($C_{k,\mathrm{decay}}$ and $C_{k,\mathrm{grow}}$ for each finite layer k, and $C_{N-1,\mathrm{decay}}$ for the last semi-infinite layer). To solve for these, $2\times N_{\mathrm{finite}}+1$ independent linear equations are needed.

At the surface (z=0, which is $z_{0,\mathrm{start}}$), the net flux leaving the tissue is related to the fluence rate

$$j_{0}(0)=-A\phi_{0}(0).$$ (12)

Here, the proportionality constant A governs the partial-current boundary condition at the tissue surface, accounting for the internal reflection caused by the refractive index mismatch between the tissue and the surrounding medium.^1^ According to Spott,^1^ A can be calculated exactly by integrating the unpolarized Fresnel reflection coefficient, $R_{\mathrm{Fresn}}(\theta )$, over the hemisphere,

$$A=\frac{1-R_{\varphi }}{2(1+R_{j})},$$ (13)

where $R_{\varphi }$ and $R_{j}$ represent the first and second angular moments of the internal Fresnel reflection, respectively. These moments are defined as^1^

$$R_{\varphi }=\int_{0}^{\pi /2}2\;\sin\;\theta \;\cos\;\theta R_{\mathrm{Fresn}}(\theta )d\theta ,$$ (14)

$$R_{j}=\int_{0}^{\pi /2}3\;\sin\;\theta {\;\cos\;}^{2}\theta R_{\mathrm{Fresn}}(\theta )d\theta .$$ (15)

The term $R_{\mathrm{Fresn}}(\theta )$ incorporates Snell’s law, averaging the s-polarized and p-polarized reflection coefficients and accounting for total internal reflection above the critical angle.

The MLDT solver evaluates these exact integrals numerically using Gaussian quadrature. For a typical tissue-to-air interface ($n_{\mathrm{tissue}}=1.4$, $n_{\mathrm{air}}=1.0$), this exact integration evaluates to A≈0.17.^1^ This rigorous partial-current approach directly balances fluxes at the physical boundary (z=0), distinguishing it from extrapolated boundary models that approximate internal reflection to define a virtual boundary plane.^2^

Substituting $\phi_{0}(0)$ and $j_{0}(0)$ (where $z_{0,\mathrm{start}}=0$),

$$\phi_{0}(0)=S_{0,0}+C_{0,\mathrm{decay}}+C_{0,\mathrm{grow}},$$ (16)

$$j_{0}(0)=-D_{0}(-S_{0,0}\mu_{\mathrm{tr},0}-\frac{C_{0,\mathrm{decay}}}{\delta_{0}}+\frac{C_{0,\mathrm{grow}}}{\delta_{0}}),$$ (17)

$$=D_{0}S_{0,0}\mu_{\mathrm{tr},0}+\frac{D_{0}}{\delta_{0}}C_{0,\mathrm{decay}}-\frac{D_{0}}{\delta_{0}}C_{0,\mathrm{grow}}.$$ (18)

Equating $j_{0}(0)=-A\phi_{0}(0)$ and rearranging

$$(\frac{D_{0}}{\delta_{0}}+A)C_{0,\mathrm{decay}}+(-\frac{D_{0}}{\delta_{0}}+A)C_{0,\mathrm{grow}}=-AS_{0,0}-D_{0}S_{0,0}\mu_{\mathrm{tr},0}.$$ (19)

By rearranging all boundary conditions into the standard linear form Ac=b, the unknown integration constants ($C_{k,\mathrm{decay}}$, $C_{k,\mathrm{grow}}$) are organized into the vector c. The coefficients of these unknowns are mapped to the matrix A, whereas all constant source terms ($S_{0,k}$) and physical parameters are moved to the vector b. Equation (19) thus forms the first row of this system.

For each internal interface k (between layer k and layer k+1, at absolute depth $z_{\mathrm{int}}=z_{k+1,\mathrm{start}}$, for k from 0 to $N_{\mathrm{finite}}-1$), two conditions must be satisfied. First, the fluence rate must be continuous across the interface ($\phi_{k}(z_{\mathrm{int}})=\phi_{k+1}(z_{\mathrm{int}})$),

$$(e^{-(z_{\mathrm{int}}-z_{k,\mathrm{start}})/\delta_{k}})C_{k,\mathrm{decay}}+(e^{(z_{\mathrm{int}}-z_{k,\mathrm{start}})/\delta_{k}})C_{k,\mathrm{grow}}\;-(e^{-(z_{\mathrm{int}}-z_{k+1,\mathrm{start}})/\delta_{k+1}})C_{k+1,\mathrm{decay}}-(e^{(z_{\mathrm{int}}-z_{k+1,\mathrm{start}})/\delta_{k+1}})C_{k+1,\mathrm{grow}}\;=S_{0,k+1}e^{-\mu_{\mathrm{tr},k+1}(z_{\mathrm{int}}-z_{k+1,\mathrm{start}})}-S_{0,k}e^{-\mu_{\mathrm{tr},k}(z_{\mathrm{int}}-z_{k,\mathrm{start}})}.$$ (20)

Second, the flux must also be continuous across the interface ($j_{k}(z_{\mathrm{int}})=j_{k+1}(z_{\mathrm{int}})$),

$$-D_{k}(-\frac{1}{\delta_{k}}e^{-(z_{\mathrm{int}}-z_{k,\mathrm{start}})/\delta_{k}})C_{k,\mathrm{decay}}-D_{k}(\frac{1}{\delta_{k}}e^{(z_{\mathrm{int}}-z_{k,\mathrm{start}})/\delta_{k}})C_{k,\mathrm{grow}}\;+D_{k+1}(-\frac{1}{\delta_{k+1}}e^{-(z_{\mathrm{int}}-z_{k+1,\mathrm{start}})/\delta_{k+1}})C_{k+1,\mathrm{decay}}+D_{k+1}(\frac{1}{\delta_{k+1}}e^{(z_{\mathrm{int}}-z_{k+1,\mathrm{start}})/\delta_{k+1}})C_{k+1,\mathrm{grow}}\;=D_{k+1}S_{0,k+1}\mu_{\mathrm{tr},k+1}e^{-\mu_{\mathrm{tr},k+1}(z_{\mathrm{int}}-z_{k+1,\mathrm{start}})}-D_{k}S_{0,k}\mu_{\mathrm{tr},k}e^{-\mu_{\mathrm{tr},k}(z_{\mathrm{int}}-z_{k,\mathrm{start}})}.$$ (21)

These equations form rows 2k+1 and 2k+2 of the **A** matrix and **b** vector. If layer k+1 is the final semi-infinite layer, the $C_{k+1,\mathrm{grow}}$ terms are omitted from both expressions because the fluence must remain finite as depth increases.

The total number of unknown coefficients is $2\times N_{\mathrm{finite}}+1$. The **A** matrix will be of size $\left(2\times N_{\mathrm{finite}}+1)\times (2\times N_{\mathrm{finite}}+1\right)$, and the **b** vector will be of size $(2\times N_{\mathrm{finite}}+1)\times 1$. This system is then numerically solved for the coefficients c.

Once the coefficients c are found, the diffuse reflectance $R_{d}$ is calculated from the net flux leaving the tissue at z=0,

$$R_{d}=-\frac{j_{0}(0)}{\Phi_{t}}.$$ (22)

### 2.3 Computational Implementation

The generalized system of linear equations derived in Sec. 2.2 results in a sparse matrix **A** with a banded structure. Rather than using standard dense matrix solvers, a vectorized banded solver was implemented.^19^

The coefficients for all wavelengths are calculated simultaneously using NumPy broadcasting. The resulting system is solved using the scipy.linalg.solve_banded routine, which is optimized for diagonal-dominant systems. This approach reduces the complexity per wavelength, allowing for the calculation of high-resolution spectra in milliseconds on a standard CPU. All MLDT simulations were performed using a 12th Gen Intel(R) Core(TM) i9-12900H CPU. For performance comparison, the GPU-accelerated MCX simulations were executed on an NVIDIA GeForce RTX 3080 Ti Laptop GPU.

### 2.4 Validation Framework

Although the diffusion approximation offers significant computational advantages over Monte Carlo simulations, its accuracy is inherently limited by its underlying assumptions. It is valid only in the scattering-dominated regime ($\mu_{a}\ll \mu_{s}^{\prime }$) where the photon distribution becomes nearly isotropic. To quantify these limitations and verify the generalized derivation, the deviation of the MLDT model from gold-standard Monte Carlo simulations was evaluated across four distinct test cases.

#### 2.4.1 Systematic homogeneous sweep

First, a systematic parameter sweep was performed for a one-layer homogeneous model to calculate the error between the MLDT diffuse reflectance predictions and MCX simulations. The error was analyzed as a function of the dimensionless ratio $\mu_{a}/\mu_{s}^{\prime }$, which serves as the primary indicator of diffusion validity. Furthermore, because the diffusion approximation relies on the reduced scattering coefficient to account for anisotropy, this analysis was repeated for four distinct anisotropy factors (g=0.25,0.50,0.75,0.90).

#### 2.4.2 Representative tissue architectures

To verify the generalized derivation in biological tissue, the forward spectral predictions of the MLDT model were compared against Monte Carlo (MCX) simulations for nine distinct tissue architectures. These encompassed three one-layer homogeneous media, three two-layer skin models, and three three-layer skin models.

#### 2.4.3 Randomized multilayer error analysis

To evaluate the solver’s performance across a broader parameter space, a large-scale validation set consisting of 400 randomized tissue models was generated. This dataset included 50 unique simulations for every layer count ranging from one to eight layers (N=1 to N=8). Parameters for each layer were randomized within physically realistic ranges for human tissue.^20^^–^^23^ The layer thickness (d) for the first layer (epidermis) was varied between 0.05 to 0.15 mm, whereas subsequent finite layers were varied between 0.25 to 0.50 mm, with the final layer always remaining semi-infinite. For the physiological parameters, the blood volume fraction (B) was randomized up to 10%, tissue oxygen saturation (S) was randomized across 0% to 100%, epidermal melanin (M) extended up to 20%, and the water content (W) was fixed at 60%. The layer scattering properties were controlled such that the scattering intensity (a) was varied between 1.0 and $4.0\;{\mathrm{mm}}^{-1}$, whereas the scattering power (b) was set between 1 and 2 to mimic varying tissue types. In addition, the scattering anisotropy (g) was maintained at 0.9 for deeper layers and 0.7 for the surface layer.

#### 2.4.4 High-N gradient modeling

Last, a distinct advantage of the generalized N-layer MLDT framework is the ability to approximate continuous gradients of optical properties by discretizing the medium into fine sub-layers. To demonstrate the solver’s stability under high-N workloads, a continuously varying tissue model consisting of 100 layers was simulated, each layer being 30  μm thick.

## 3 Results

### 3.1 Systematic Homogeneous Sweep

Figure 1(a) displays the percent error in calculated diffuse reflectance as a function of the absorption-to-reduced scattering ratio. The relative error grows drastically as the medium becomes more absorbing (or less scattering). Notably, the model exhibits sensitivity to anisotropy; higher g values (typical of biological tissue, where g≈0.9) result in slightly higher deviations at extreme absorption ratios compared with more isotropic scattering (g=0.25). Figure 1(b) illustrates the absolute error for the same parameters. This distinction is crucial because large percent errors often occur in regions of very low reflectance (high absorption), where the absolute difference in signal is practically negligible for many experimental setups.

**Fig. 1:**
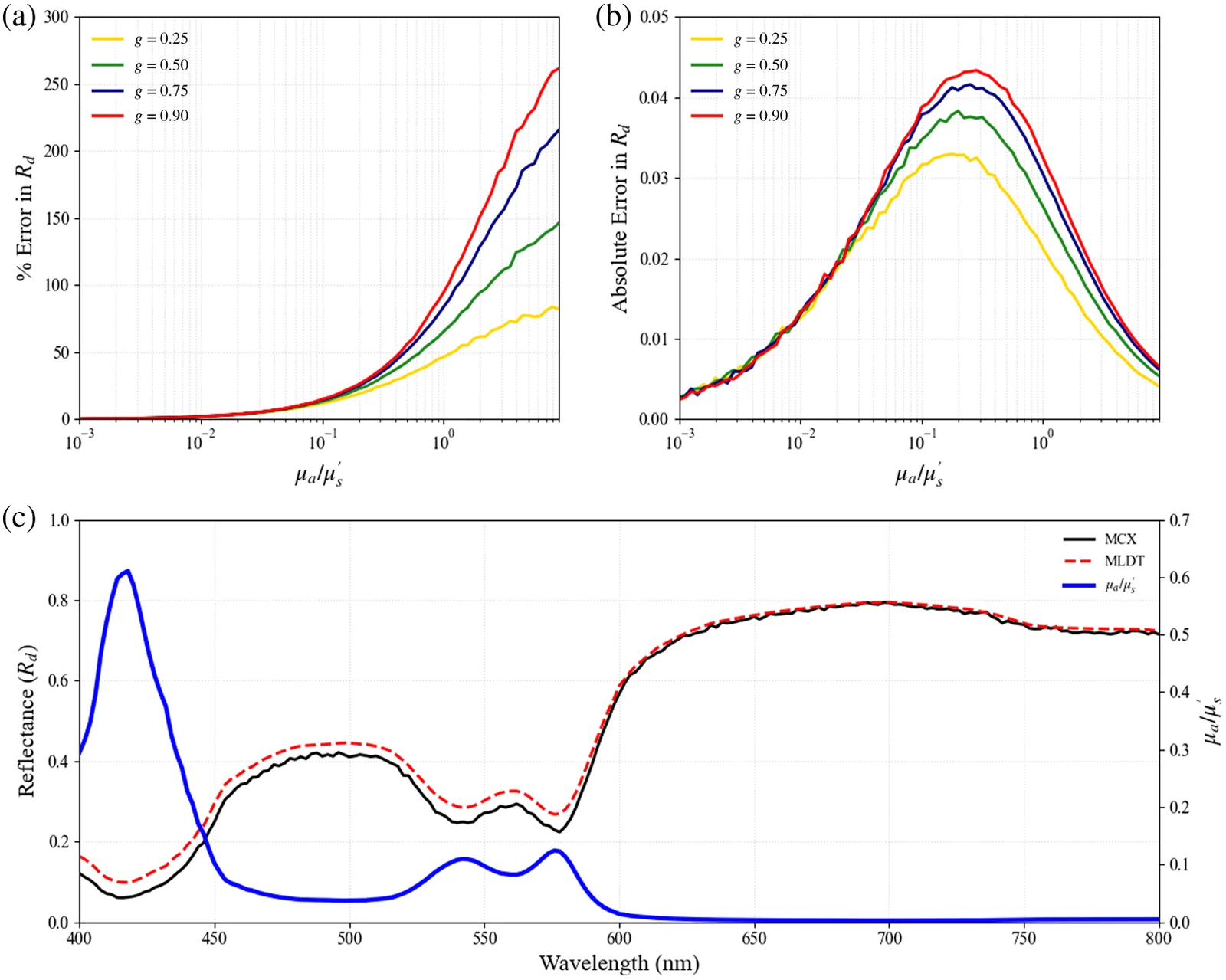
Systematic error analysis of the MLDT model compared with MCX simulations (1 million photons). (a) Percent error in diffuse reflectance ($R_{d}$) as a function of the absorption-to-scattering ratio ($\mu_{a}/\mu_{s}^{\prime }$) for anisotropy factors g=0.25,0.5,0.75, and 0.9. (b) Absolute error in diffuse reflectance ($R_{d}$; 0 to 1) as a function of the absorption-to-scattering ratio ($\mu_{a}/\mu_{s}^{\prime }$) for anisotropy factors g=0.25,0.5,0.75, and 0.9. (c) Comparison of forward model spectra ($R_{d}$ versus wavelength) for a representative homogeneous biological tissue model plotted alongside the $\mu_{a}/\mu_{s}^{\prime }$ ratio. The rapid increase in this ratio below 450 nm correlates directly with the increased error observed between the MLDT prediction (red dashed line) and MCX simulation (black solid line).

To contextualize these systematic errors within a realistic application, Fig. 1(c) applies the model to a homogeneous representative biological tissue. Figure 1(c) shows the spectral overlay of the MLDT prediction against the MCX simulation from 400 to 800 nm. The agreement is excellent in the red and near-infrared (NIR) regions but deviates in the visible range. Figure 1(c) plots the $\mu_{a}/\mu_{s}^{\prime }$ ratio for this specific tissue model. By correlating Fig. 1(c) with Fig. 1(a), it becomes evident that the spectral deviation below 550 nm is driven by the sharp rise in hemoglobin and melanin absorption, which pushes the ratio beyond the diffusion limit of ≈0.1. This analysis establishes that although the MLDT solver is highly accurate for the wavelengths above ∼580  nm, corresponding to the therapeutic window, users must exercise caution when analyzing the blue-green spectral region where the diffusion assumption is challenged, as is consistent with conventional use of the diffusion approximation.

### 3.2 Representative Tissue Architectures

Figure 2 presents spectral overlays for the nine distinct tissue architectures involving one, two, and three layers. The optical properties were chosen to represent realistic tissue ranges. As observed, the MLDT solver (red dashed lines) captures the spectral features of the MCX simulations (black solid lines) with high fidelity across the NIR window and expected minor deviations appearing in the visible range (<500  nm) due to high absorption.

**Fig. 2:**
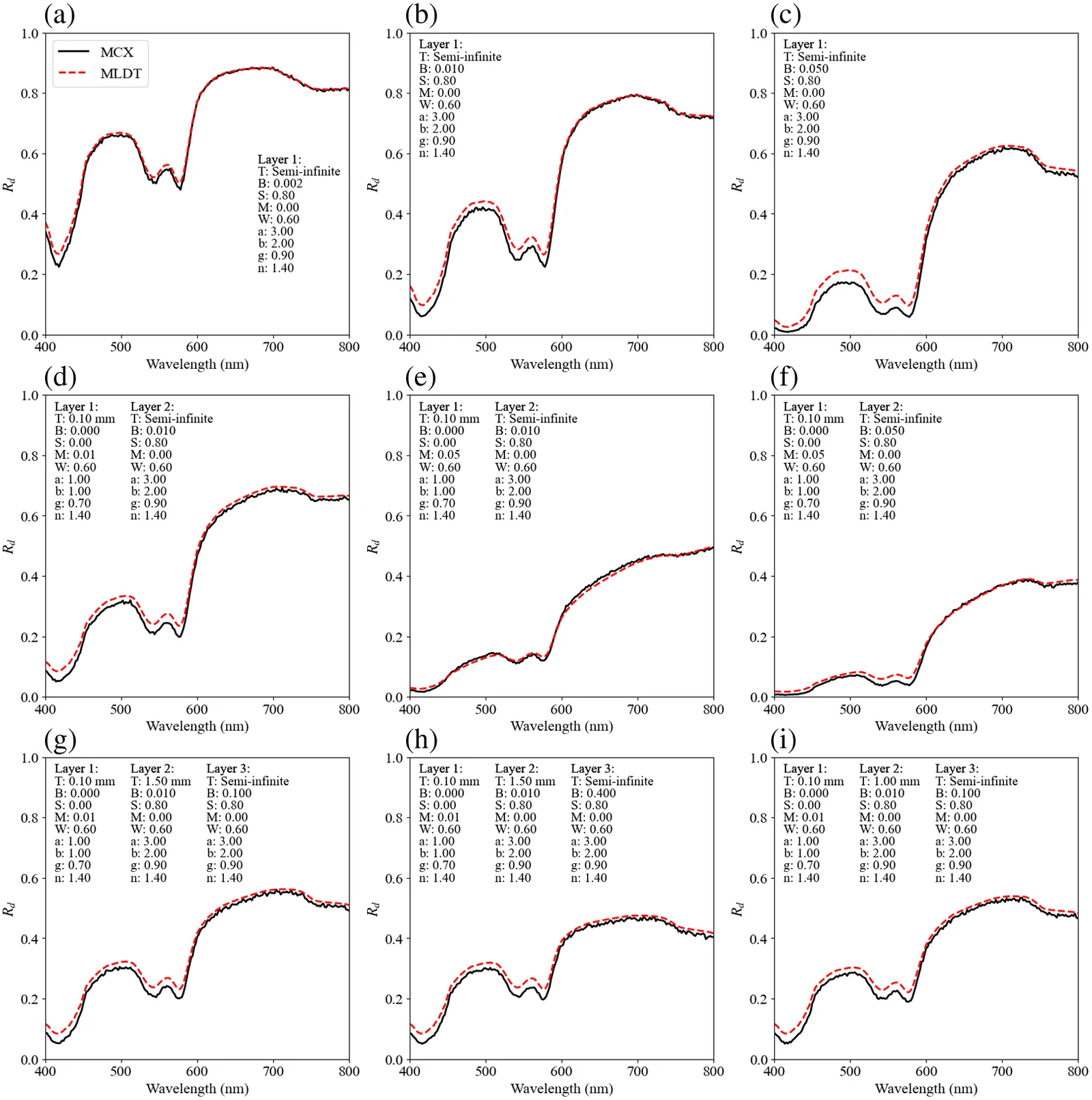
Comparison of forward model spectra generated by the MLDT solver (red dashed lines) and MCX simulations (black solid lines). The figure presents nine representative simulations organized by tissue complexity: (a)–(c) One-layer homogeneous models, (d)–(f) two-layer models, and (g)–(i) three-layer models.

### 3.3 Large-Scale Randomized Validation

To evaluate the solver’s robustness across a vast parameter space, 400 randomized simulations (50 per layer count, N=1 to 8) were analyzed. This large-scale test was designed to quantify how error scales with structural complexity and to evaluate the computational efficiency of the matrix-based approach.

Utilizing the vectorized banded matrix approach, the MLDT solver computed the entire 400-simulation validation set in 3.49 s on a standard consumer CPU. The mean MLDT computation time was 0.0087 s per spectrum, compared with a mean of 2424.95 s for the GPU-accelerated MCX simulations. This represents a total speedup of ∼277,607× over the gold-standard stochastic method.

The global accuracy of the solver is summarized in Fig. 3. Figures 3(a) and 3(c) display the aggregate mean absolute error (MAE) and mean absolute percent error (MAPE) across all 400 simulations, respectively. The global MAE remains below 0.017 $R_{d}$ units across the entire spectrum, with the median spectral root mean square error (RMSE) across the dataset found to be 0.0145.

**Fig. 3:**
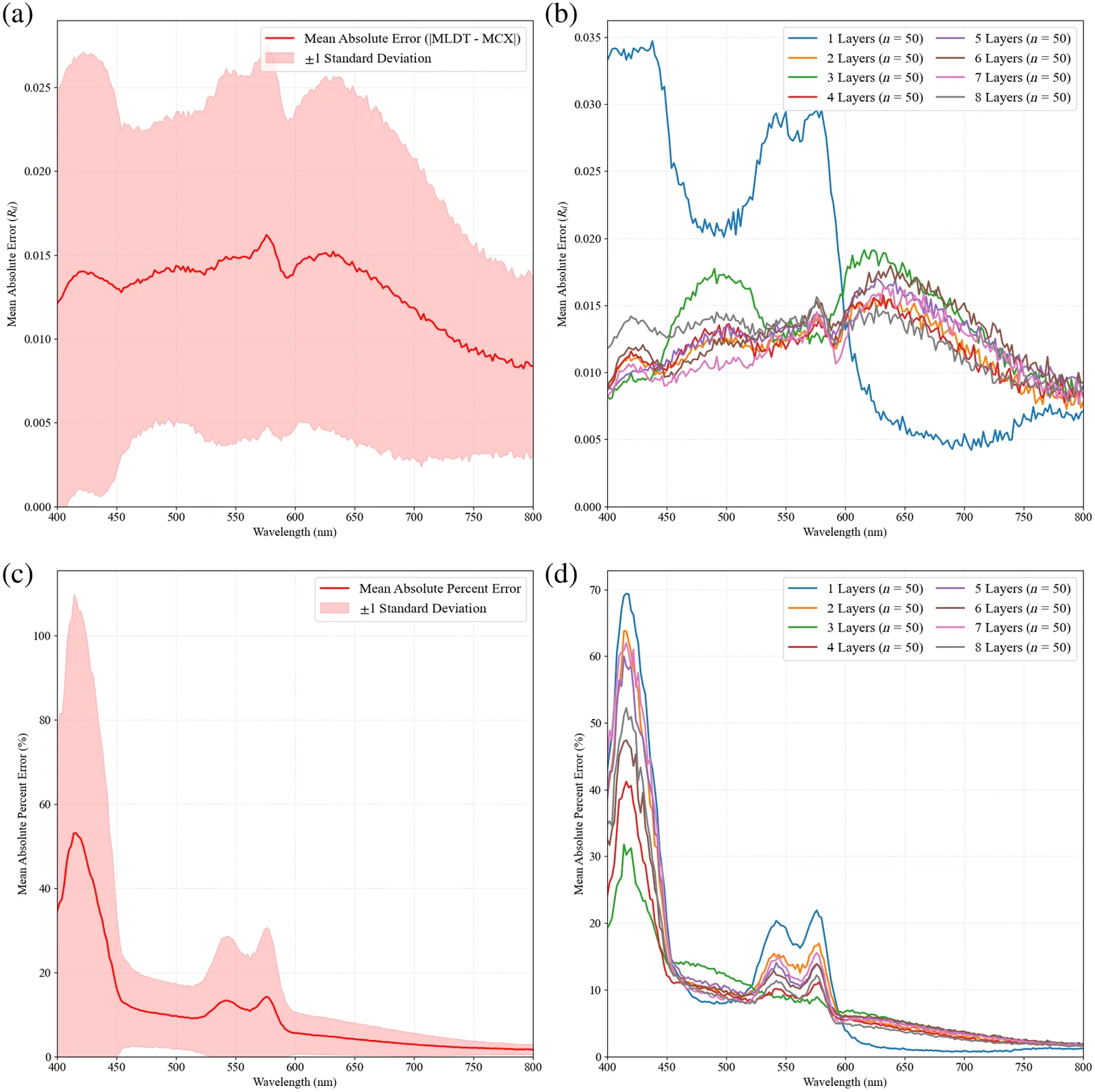
Statistical performance of MLDT across 400 randomized simulations (N=1 to 8). (a) Aggregate mean absolute error (MAE) with shaded ±1 standard deviation. (b) MAE broken down by layer count, demonstrating that error does not scale with structural complexity. (c) Aggregate mean absolute percent error (MAPE) with shaded ±1 standard deviation. (d) MAPE broken down by layer count, demonstrating that error does not scale with structural complexity.

Crucially, as shown in Figs. 3(b) and 3(d), the error does not scale with structural complexity. The absolute residuals for high-N architectures are comparable to, and in some spectral regions lower than, those of simpler models. For example, the one-layer homogeneous models exhibit the highest mean absolute residuals in the visible spectrum (400 to 600 nm), peaking near 0.035 $R_{d}$ units. By contrast, the multilayer models (N=2 to 8) exhibit clustered residuals of lower magnitude in the same range. This demonstrates that the recursive matrix approach maintains high numerical stability regardless of the number of internal interfaces.

### 3.4 High-*N* Gradient Modeling

Figure 4 illustrates the depth-dependent optical profiles used for a 100-layer simulation. Unlike standard step-function models, this simulation incorporates continuous Gaussian distributions of blood volume fraction (B) and scattering intensity (a) and a localized, sharp peak of melanin concentration (M) near the surface, simulating a discrete pigmented layer such as the basal layer or a nevus. Despite the increase in system size to a 201×201 matrix, the solver computed the full spectrum (400 to 800 nm, 2 nm resolution) in less than 70 ms on a standard consumer CPU.

## 4 Discussion

The results presented in this work demonstrate that the MLDT solver effectively bridges the gap between accurate but computationally expensive Monte Carlo simulations and efficient but over-simplified homogeneous analytical models. By accurately modeling the stratified nature of tissue, the MLDT approach allows for the quantitative separation of chromophores located at different depths, a capability that is critical for determining the physiological status of biological tissues.

The primary motivation for developing a generalized analytical N-layer model is computational speed. Although Monte Carlo simulations provide the gold-standard for accuracy by rigorously solving the RTE, they are stochastic and computationally intensive. In the context of inverse problems, where a forward model may be called thousands of times to fit a single spectrum, Monte Carlo is often impractical for real-time clinical applications. Utilizing the vectorized banded matrix approach, the MLDT solver demonstrated a mean computation time of 0.0087 s per spectrum on a standard CPU. This represents a speedup of ∼277,607× compared with GPU-accelerated Monte Carlo simulations, which averaged over 40 min per spectrum. Such performance enables the use of robust iterative optimization algorithms to extract depth-resolved optical properties in real time.

A critical finding of the large-scale randomized validation is that spectral error does not scale with structural complexity (Fig. 3(b)). Unlike numerical methods where errors may accumulate at each layer interface, the MLDT recursive matrix formalism maintains high numerical stability across an arbitrary number of stratified layers. Interestingly, the one-layer homogeneous models exhibited the highest mean absolute residuals in the visible spectrum (400 to 600 nm), whereas multilayer models (N=2 to 8) maintained lower, clustered error profiles. This is due to the lack of a blood-free epidermis in the one-layer homogeneous model. These results suggest that the generalized solver is as robust for complex stratified architectures as it is for simple ones, making it a viable tool for discretized gradient modeling (Fig. 4).

**Fig. 4:**
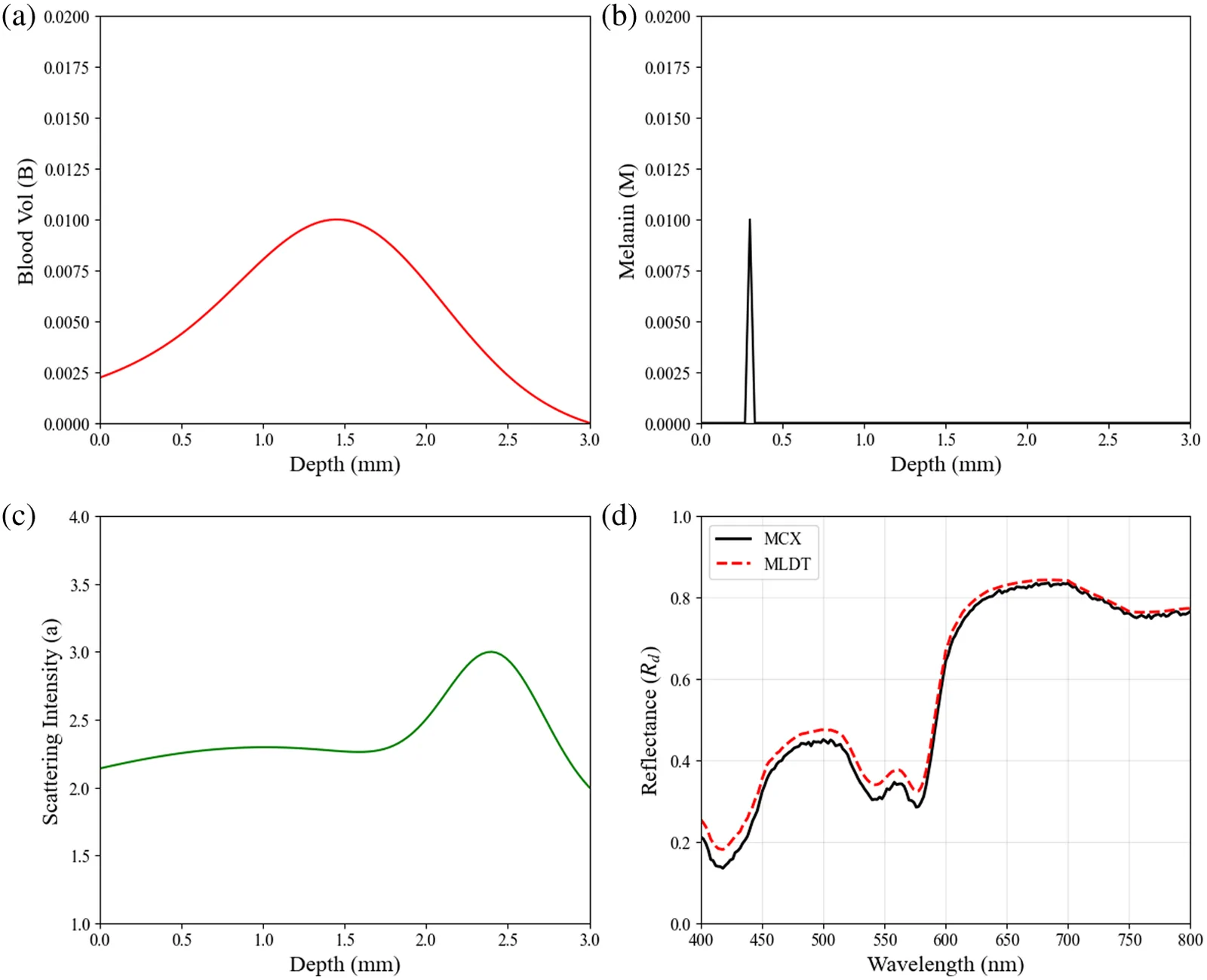
Demonstration of high-resolution depth profiling with N=100 layers. The model inputs vary continuously with depth: (a) blood volume fraction (B) follows a Gaussian distribution; (b) melanin concentration (M) exhibits a sharp, localized peak; and (c) scattering amplitude (a) follows a smooth gradient. (d) The resulting diffuse reflectance spectrum. The MLDT solver (red dashed line) maintains excellent agreement with the MCX simulation (black solid line), validating the solver’s capability to model complex, heterogeneous tissue architectures.

Despite its utility, the MLDT model is bound by the fundamental limitations of diffusion theory. As shown in Figs. 1 and 3(c), accuracy degrades in the visible range below 500 nm, where high absorption by hemoglobin and melanin pushes the $\mu_{a}/\mu_{s}^{\prime }$ ratio beyond the diffusion limit. Diffusion theory assumes scattering dominance ($\mu_{s}^{\prime }\gg \mu_{a}$) and near-isotropic fluence; in high-absorption regimes, the P1 approximation fails to accurately predict the rapid attenuation of light near the surface. Consequently, although the MLDT model is highly accurate for red and NIR windows (600 to 1000 nm), care must be taken when analyzing signals in the blue-green spectrum. For applications requiring high accuracy in high-absorption regimes, a hybrid approach combining initial Monte Carlo steps with a diffusion tail may represent an optimal compromise.

Although the current MLDT solver utilizes the P1 approximation to prioritize computational speed, recent advancements have demonstrated that higher-order spherical harmonics, such as the P3 approximation, can further improve accuracy near boundaries and in high-absorption regimes.^6^ Extending the current matrix framework to accommodate the coupled differential equations of the P3 approximation represents a promising direction for future open-source development. Furthermore, the present formulation is restricted to a 1D depth-dependent fluence rate, effectively modeling an infinitely broad collimated beam. To account for the source-detector separation distance in spatially resolved reflectance measurements, future iterations of the solver will integrate a Hankel transform approach to solve the cylindrical diffusion equation in the spatial frequency domain.

## 5 Conclusion

In this study, a comprehensive derivation and open-source Python implementation of a generalized multilayer diffusion theory (MLDT) model was presented. The model was validated against 400 randomized Monte Carlo simulations across N=1 to eight layers, demonstrating excellent agreement in the diffusion regime with a median spectral RMSE of 0.0145.

The generalized N-layer framework allows for the flexible modeling of complex tissue architectures without the restrictive geometry assumptions of previous analytical solutions. By providing this tool as an open-source resource, we aim to facilitate the development of real-time, quantitative spectroscopic systems capable of probing the stratified complexity of biological tissue.

## Data Availability

All relevant code, data, and materials are available at https://github.com/ttlivecchi/mldt.git.
